# The effects of emotional exhaustion and resilience on the relationship between the practice environment and turnover intentions among acute care nurses: A moderated mediation analysis

**DOI:** 10.1016/j.ijnsa.2026.100521

**Published:** 2026-03-16

**Authors:** Elizabeth H. Fraser, Lingling Zhang, Anna E.Schierberl Scherr, Sun S. Kim

**Affiliations:** a Manning College of Nursing and Health Sciences, University of Massachusetts Boston, Boston, MA, United States; b Department of Psychology, University of Massachusetts Dartmouth, Dartmouth, MA, United States; c South Shore Hospital, South Weymouth, MA, United States

**Keywords:** Organizational and workforce issues, Nurses, Emotional exhaustion, Burnout, Resilience, Turnover intentions

## Abstract

**Background:**

Nursing shortage is a complex national crisis compounded by high turnover of qualified nurses, particularly during and in the post-pandemic period.

**Objectives:**

To examine the effects of emotional exhaustion and resilience on the relationship between the practice environment and turnover intentions among acute care nurses.

**Design:**

Cross-sectional descriptive online survey study.

**Setting (s):**

Acute care hospital settings in the United States.

**Participants:**

A total of 240 acute care nurses were recruited between October 2023 and April 2024.

**Methods:**

Participants completed four validated instruments: Practice Environment Scale-Nursing Work Index, Workplace Resilience Scale, Maslach Burnout Inventory-Human Service Survey for emotional exhaustion, and Anticipated Turnover Scale. Data were analyzed using moderated mediation regressions with Hayes’ PROCESS macro in SPSS version 28.0.

**Results:**

Emotional exhaustion mediated the relationship between practice environment and turnover intentions (*B* = −0.79, 95% CI [−1.22, −0.35], *p* < .001). Resilience moderated the pathway between practice environment and emotional exhaustion (*B* = 9.81, 95% CI [1.89, 17.73], *p* = .015), such that the association was stronger among nurses with lower resilience. The model explained 30% of the variance in turnover intentions.

**Conclusions:**

Emotional exhaustion is a central mechanism linking practice environment conditions to turnover intentions. Resilience functions as a preventative moderator, buffering the impact of practice environment on emotional exhaustion.

**Implications for clinical practice:**

The findings support multilevel strategies that address both work conditions and resilience to promote retention among acute care nurses.

**What is already known**

• Emotional exhaustion predicts nurses’ turnover intentions.
• Practice environment influences emotional exhaustion and retention in acute care nurses.
• Resilience may buffer workplace stress, but its stage-specific role in turnover pathways is unclear.

Alt-text: Unlabelled box dummy alt text

**What this paper adds**

• Emotional exhaustion mediates the link between practice environment and turnover intentions.
• Resilience moderates the practice environment–emotional exhaustion pathway, but not the exhaustion–turnover pathway.
• Staffing and resource adequacy show the strongest associations with emotional exhaustion, particularly among nurses with lower resilience.
• Retention strategies should address both work conditions and resilience to prevent emotional exhaustion and reduce turnover intentions.

Alt-text: Unlabelled box dummy alt text

## 1 Introduction

Registered nurses (RN) represent the largest sector of the United States (U.S.) healthcare workforce, with 3.3 million employed, and more than half (58 %) working in hospitals, yet the nation continues to face a critical nursing shortage intensified by the outbreak of coronavirus disease 2019 (COVID-19; Bureau of Labor Statistics, 2025). Turnover is a primary driver of this shortage, defined as leaving an organization for another nursing position or exiting the profession entirely, and needs to be replaced (Bureau of Labor Statistics, 2025; Kovner et al., al., 2014). The high turnover rate in hospitals incurs an average annual cost of $4.75 million, with each RN replacement averaging $61,110 (Colosi, 2025).

One factor that has gained increasing attention is resilience, which may play a role in nurse retention, as it is critical for overcoming stress and hardships at work (Delgado et al., 2020, 2021; Dyrbye et al., 2019; Gao et al., 2017). Equally important is the construct of emotional exhaustion, the core dimension of burnout, which arises when prolonged on-the-job stress goes unmanaged (Maslach et al., 2018). Emotional exhaustion has been linked to reduced job satisfaction, diminished well-being, and increased turnover intentions among nurses in acute care settings (Kelly et al., 2021; Maslach et al., 2018; Rushton et al., 2015). There is a paucity of studies on how both personal factors and environmental factors are related to resilience and emotional exhaustion, and how these variables together influence turnover intentions. Accordingly, this study examines the complex relationship among the practice environment (i.e., hospital settings), emotional exhaustion, and resilience, and their impact on turnover intentions among acute care nurses.

This study is grounded in the Neuman Systems Model (1982), in which resilience functions as a flexible line of defense that buffers stress; when weakened, it increases nurses' vulnerability to burnout (Maslach et al., 2018) and turnover intentions (Marsh et al., 1999; Turner and Kaylor, 2015). Research on nurse turnover has increasingly emphasized the importance of not only identifying predictors but also clarifying the mechanisms and conditions under which turnover intentions develop. This study employed a moderated mediation analysis design to address these questions. Drawing on the Neuman’s Systems Model, emotional exhaustion was conceptualized as a key mediating pathway that may help explain how practice environment relates to turnover intentions. In addition, resilience level was incorporated as a moderating variable, reflecting its role as a personal resource that may mitigate the impact of practice environment on turnover intentions.

The nursing practice environment shapes nurse job outcomes and patient care quality, making it a priority for nurse leaders to measure and enhance its characteristics (Lake, 2002). U.S. nurses who left their jobs and those who considered leaving cited stressful practice environment and burnout as primary reasons (Shah et al., 2021). Emotional exhaustion is considered the strongest characteristic of burnout (Maslach et al., 2018). Nurses are required to perform patient care promptly and accurately while navigating competing priorities in a high-stress environment, which can contribute to job burnout, a psychological symptom in response to chronic on-the-job stressors (Maslach et al., 2018; Rangachari and L. Woods, 2020). Exhausted and stressed nurses are more likely to make poor decisions at work and exhibit changes in bedside manner, such as insensitivity and lack of empathy, ultimately impacting patient outcomes (The Joint Commission, 2019).

Resilience is broadly defined as a set of personal characteristics and an ongoing, interactive dynamic process that enables individuals to adapt to adversity within the work environment (Delgado et al., 2021); it acts as a protective factor for acute care nurses against stress and emotional exhaustion (Kim et al., 2019; Vala et al., 2024; Zhang et al., 2021). Resilient acute care nurses demonstrate better responses to workplace stress, effectively controlling the harmful effects of feeling overwhelmed or burnt out (Delgado et al., 2020, 2021; Gao et al., 2017; Rushton et al., 2015) and greater intent to stay in the profession, even during the COVID-19 pandemic (Aiken et al., 2022; Kelly et al., 2021). In 2021, the National Academies of Sciences, Engineering, and Medicine ([NASEM], 2021) prioritized nurses’ well-being in their report*, The Future of Nursing 2020–2030*. This report suggests that resilience is essential for nurses working in complex environments.

### 1.1 Study aim

This study examined how the practice environment, emotional exhaustion, and resilience influence turnover intentions among acute care nurses. First, a mediation analysis was performed to assess the mediating effect of emotional exhaustion on the relationship between the practice environment and turnover intentions among acute care nurses. Second, a moderated mediation analysis was conducted to test the hypothesis that resilience moderates the relationship between the practice environment and emotional exhaustion, with emotional exhaustion mediating the effect of the practice environment on acute care nurses’ turnover intentions.

## 2 Methods

### 2.1 Study design and data source

The study employed a cross-sectional descriptive design, conducting an online survey between October 2023 and April 2024. Acute care nurses were the target population. Initially, a sample of registered nurses was generated from the Board of Nursing using a publicly accessible list in 11 northeastern states in the US. People were asked to participate in the survey if they were nurses currently working in an acute care setting. Due to low response rates and financial constraints, the study expanded to include a convenience sample of acute care nurses recruited through social media platforms (e.g., Facebook, LinkedIn, and nursing association websites). Inclusion criteria were registered nurses who worked in an acute care environment and provided direct patient care for a minimum of 50 % of their work time. Newly graduated nurses who had worked for less than three months were excluded.

Using the G*Power software (latest ver. 3.1.9.7; Heinrich-Heine-Universität Düsseldorf, Düsseldorf, Germany), a sample size of 178 was required for the proposed mediation and moderation analyses with three predictors to have 90 % of the power with a two-sided alpha level of 0.05. There was no prior study testing the proposed relationship, and therefore, the sample size calculation was based on the effect size of $f^{2}\;$=0.06, that is between low and medium effect sizes. To account for possible missing values at a rate of 20 %, the final target sample size was estimated to be at approximately 214 participants.

### 2.2 Measures

The practice environment was measured using the Practice Environment Scale of the Nursing Work Index (Lake, 2002). This 31-item self-report questionnaire contains five subscales: (a) nurse participation in hospital affairs, (b) nursing foundations for quality of care, (c) nurse management, leadership, and support of nurses, (d) staffing and adequate resources, and (e) collegial nurse-physician relationships. Each item is rated on a 4-point scale (1=*strongly disagree*, 4= *strongly agree*), and each scale score is the average score. The overall composite score can be calculated as the average of the five subscales scores. A higher score portrays more agreement that the question items are present in the acute care nurse's current professional status. Cronbach’s alpha for the composite score was 0.93 in this study.

Resilience was measured by the Workplace Resilience Scale. This 20-item self-report questionnaire has four subscales: (a) active problem-solving, (b) team efficacy, (c) confident sensemaking, and (d) bricolage (Mallak andYildiz, 2016). The items are rated on a 5-point scale from 1 (*not true at all*) to 5 (*true nearly all the time*). The overall composite score is calculated by averaging the scores from each of the four subscales. The higher the scores are, the greater the talent is in each dimension. Cronbach’s alphas across the four subscales and the composite score were. 75 to 0.84 and 0.89, respectively in this study.

Emotional exhaustion was measured using the Maslach Burnout Inventory Scale-Human Services Survey (Maslach et al., 2018). This 9-item self-report questionnaire assesses feelings of being emotionally drained by one’s job and uses a 7-point frequency scale ranging from 0 (*never*) to 7 (*every day*). The three subscales: (a) emotional exhaustion, (b) depersonalization, and (c) lack of personal accomplishment were scored using the SUM Method, where item responses are added and interpreted separately to produce independent subscale scores. The higher scores correspond to more significant experienced burnout. In this study, Cronbach’s alpha of the scale was 0.92.

Turnover intentions were measured by the Anticipated Turnover Scale (ATS; Hinshaw et al., 1985). This 12-item self-report questionnaire is rated on a 7-point Likert scale (1=*strongly disagree*, and strongly do not intend to leave; 7=*strongly agree*, and strongly intend to leave). Six of the 12 items are worded in opposition to the others and hence, require reverse coding. Its Cronbach’s alpha was 0.89 in this study.

### 2.3 Data analysis

Data analysis was performed using the International Business Machine (IBM) Statistical Package for Social Sciences (SPSS) Version 28. Prior to conducting descriptive and inferential statistical analyses, all study variables were examined for distribution, missing values, and outliers. Normality was assessed using histograms, Q-Q plots, skewness, and kurtosis statistics. Continuous variables were summarized using means and standard deviations. Categorical variables were summarized using frequencies and percentages. Correlation analysis examined relationships between variables using Pearson's correlation coefficients for continuous variables and Spearman's *rhos* for categorical variables to assess associations and confirm the absence of multicollinearity prior to the analysis. The *p*-value ≤ 0.05 was considered statistically significant.

Multiple linear regression analyses were performed to identify significant predictors of turnover intentions. Then, a moderated mediation analysis was conducted using Hayes (2022) PROCESS macro add-on to SPSS (Model 7) with 5000 bootstrap samples. First, the indirect effects of the mediator (emotional exhaustion) were estimated by resampling the data 5000 times to create a distribution of possible indirect effects from 95 % bootstrap confidence intervals (CI; Hayes, 2022). Second, moderated mediation analysis estimated the significance of the moderation indirect effect at different levels (low resilience versus high resilience) on the relationship between the practice environment and turnover intentions (Hayes, 2022). An index of moderation was used to test the significance of the moderated mediation, for example, the difference in the indirect effects between high and low levels of resilience, using the median score (Hayes, 2022). Sensitivity analysis was also conducted to detect the moderation effect of resilience on the relationship between emotional exhaustion and turnover intentions.

### 2.4 Ethical consideration

Prior to recruitment, this study was reviewed and approved by the University of Massachusetts Boston IRB (#3545). Participants were offered the option to enter a raffle for a $100 electronic gift card as an incentive for completing the survey.

## 3 Results

Among 240 participants, 105 were randomly selected and their response rates were 2 % and 1 % for postal mail and email, respectively. Preliminary analyses showed no significant differences in demographics or study variables between the two samples, and therefore, combined results are presented (Table 1). The majority of the participants identified as female (*n* = 228, 95.00 %) and non-Hispanic/Latino (*n* = 231, 96.30 %), with most being white (*n* = 218, 90.80 %). More than half of them were married/cohabiting with a significant other (*n* = 154, 64.20 %), followed by those never married (*n* = 64, 26.70 %). The majority of nurses had a baccalaureate degree (*n* = 164, 68.30 %), and most worked full-time (*n* = 165, 68.8 %). The average years of nursing practice were 14.26 ± 11.73 years, and in their current job, 7.06 ± 7.95 years. The two most frequently reported clinical settings were adult medical-surgical/telemetry (*n* = 123, 51.20 %) and critical care/step-down (*n* = 101, 42.10 %).

**Table 1:** Demographic characteristics (*N* = 240).

Variables	*n* ( %) or Mean ± SD
Sex	
Male	12 (5.00)
Female	228 (95.00)
Age	41.28 ± 12.93
Ethnicity	
Hispanic/ Latino	9 (3.80)
Non-Hispanic/Latino	231 (96.30)
Race	
White	218 (90.80)
Black or African American	7 (2.90)
Asian	12 (5.00)
Some other Race	3 (1.30)
Marital Status	
Never Married	64 (26.70)
Married/Cohabitating	154 (64.20)
Widowed	1 (0.40)
Divorced/Separated	21 (8.80)
Highest Nursing Degree	
Diploma	10 (4.20)
Associate’s	31 (12.90)
Bachelor’s	164 (68.30)
Master’s	30 (12.50)
Doctorate	5 (2.10)
Job Situation	
Full-time	165 (68.80)
Part-Time	40 (16.70)
Per Diem	35 (14.60)
Years as Registered Nurse	14.26 ± 11.73
Years in Current Job	7.06 (7.95)
Unit Type	
Adult CCU/Step-Down	101 (42.10)
Adult M-S/Tele	123 (51.20)
Pediatric M-S/Tele	16 (6.70)
Resilience	3.88 (0.47)
Practice Environment	2.75 (0.43)
Emotional Exhaustion	28.60 (12.73)
Turnover Intentions	3.58 (1.32)

SD = standard deviation; M-S/Tele= Medical- Surgical/.

Telemetry.

### 3.1 Descriptive statistics and bivariate associations

Staffing and resource adequacy were rated lowest among the practice environment subscales (2.46 ± 0.63), followed by hospital affairs (2.69 ± 0.50) and nurse manager leadership (2.76 ± 0.61). Nursing foundations (2.90 ± 0.42) and nurse–physician relations (2.93 ± 0.55) were rated more favorably than the other three subscales. When examined by resilience group (median split), the association between staffing and resource adequacy and emotional exhaustion was stronger among nurses with lower resilience (*r* = –.623, *p* < .001, *n* = 119) compared to those with higher resilience (*r* = –.370, *p* < .001, *n* = 121).

Bivariate analyses indicated that all practice environment subscales were significantly negatively correlated with emotional exhaustion. Staffing and resource adequacy demonstrated the strongest association (*r* = –.520, *p* < 0.001), followed by nurse manager leadership (*r* = –.427, *p* < 0.001), hospital affairs (*r* = –.388, *p* < 0.001), nursing foundations (*r* = –.340, *p* < 0.001), and nurse–physician relations (*r* = –.224, *p* < 0.001).

### 3.2 Multivariable regression analysis

Results of a multivariable regression analysis indicated that the overall model was statistically significant, *F*(3, 236) = 35.26, *p* < .001, explaining 30 % (Adjusted R^2^ = 0.301) of the variance in turnover intention (Table 2). Regarding individual predictors, emotional exhaustion was the most significant unique contributor (*B* = 0.04, 95 % CI [0.03, 0.05], *p* < .001), followed by practice environment (*B* = −0.76, 95 % CI [−1.15, −0.37], *p* < .001). Nurses with high levels of emotional exhaustion had higher turnover intentions. The relationship between resilience and turnover intentions was insignificant when the practice environment and emotional exhaustion were controlled.

**Table 2:** Multivariable linear regression predicting turnover intentions (*n* = 240).

Variable	*B*	*SE*	*t*	*p*	95 % CI
Practice Environment	−0.76	0.20	−3.85	<0.001	−1.15, −0.37
Resilience	−0.09	0.17	−0.55	.584	−0.42, 0.23
Emotional Exhaustion	0.04	0.01	6.00	<0.001	0.03, 0.05

*B* = Unstandardized regression coefficient; SE = Standard error of the coefficient;.

CI=Confidence intervals.

### 3.3 Moderated mediation model

**Fig. 1:**
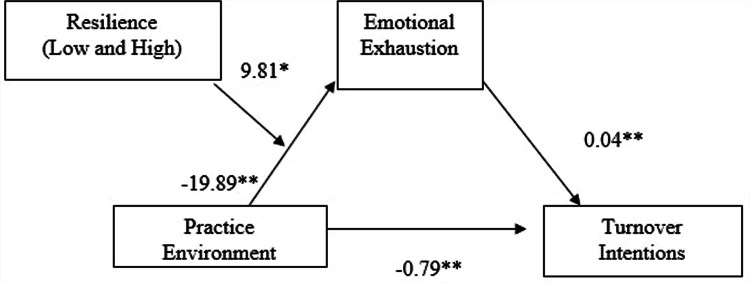
Statistical model of moderated mediation (*N* = 240). Conditional indirect effects of acute care nurses’ practice environment on turnover intentions through emotional exhaustion at low and high levels of resilience. The moderation effect represents the interaction between practice environment and resilience predicting emotional exhaustion. All presented effects are unstandardized regression coefficients. *p < .05, **p < .001.

The model showed a significant effect of practice environment on turnover intentions (*B* = −1.37, 95 % CI [−1.72, −1.01], *p* < .001), which was reduced but remained significant when emotional exhaustion was included in the model (*B* = −0.79, 95 % CI [−1.22, −0.35], *p* < .001), demonstrating partial mediation (Fig. 1, Table 3).

**Table 3:** Conditional process analysis (*N* = 240).

Variable	Emotional Exhaustion				Turnover Intentions			
	*B*	*SE*	95 % CI	*p*	*B*	*SE*	95 % CI	*p*
Practice Environment	−19.89	3.13	−26.05,−13.73	<0.001	−0.79	0.22	−1.22, −0.35	<0.001
Resilience (Ref=low resilience)	−2.65	1.52	−5.66, 0.35	.083				
Practice Environment x Resilience	9.81	4.02	1.89, 17.73	.015				
Emotional Exhaustion					0.04	.01	0.03, 0.05	<0.001
Constant	29.39	1.04	27.33, 31.44	<0.001	2.45	0.23	2.00, 2.89	<0.001

*B*= unstandardized regression coefficient; *SE*= standard error; CI = confidence interval.

Model for emotional exhaustion as a mediator *R*^2^ = 0.28, *F* (3, 236) =27.02, *p* < .001.

Model for turnover intention as the dependent variable *R*^2^ = 0.31, *F*(2, 237)= 52.37, *p* <. 001.

**Fig. 2:**
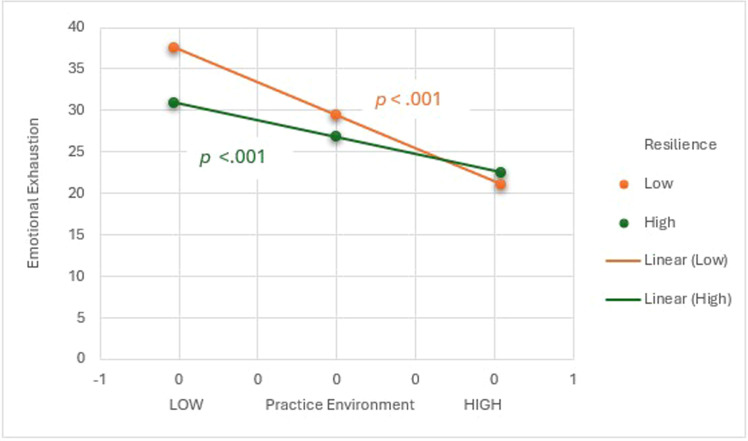
Simple slopes demonstrating the interaction of moderation analysis.

There was a significant interaction effect between practice environment and resilience in predicting emotional exhaustion, *B* = 9.81, 95 % CI [1.88, 17.73], *p* = .015, Δ*R*^2^ = 0.023 (Table 3). As shown in Fig. 2, the negative conditional effect from the practice environment on emotional exhaustion was strongest for low levels of resilience, *B* = −19.89, 95 % CI [−26.05, −13.73], *p* < .001; it was weaker but still significant for high values, *B* = −10.08, 95 % CI [−15.06, −5.10], *p* < 0.001. Acute care nurses with lower resilience and less favorably rated practice environments reported higher emotional exhaustion than those with higher resilience. In contrast, resilience did not moderate the relationship between emotional exhaustion and turnover intentions, as the interaction term between emotional exhaustion and resilience was not significant (*F*(1, 235) = 1.14, *p* = .287). These findings indicate that resilience moderated the first stage of the mediation pathway (practice environment → emotional exhaustion) but not the second stage (emotional exhaustion → turnover intentions).

## 4 Discussion

### 4.1 Main findings and theoretical interpretation

The finding that the practice environment demonstrated a significant direct effect on turnover intentions is consistent with previous research conducted in the U.S. (Gensimore et al., 2020), Canada (Boudreau and Rheaume, 2024), and Malaysia (Ying et al., 2020), underscoring the universal importance of a supportive work environment for nurse retention. Acute care nurses work on the frontline in high-demand clinical settings and must continually navigate complex and stressful conditions (Martin et al., 2023). This study also found that emotional exhaustion mediated the relationship between practice environment and turnover intentions and that resilience moderated the pathway between practice environment and emotional exhaustion. The mediating role of emotional exhaustion supports recent work by Boudreau and Rheaume (2024) in acute care settings. Prior research also identified resilience as a moderator in the relationship between practice environment and turnover intentions (Gensimore et al., 2020), although the present study further specifies the stage at which this moderating effect occurs. While the moderating role of resilience in this relationship has been reported, this study extends the literature by clarifying the mechanisms through which the practice environment influences turnover intentions and distinguishing the point in the pathway at which resilience exerts its strongest influence.

Consistent with the previous study, emotional exhaustion mediated the relationship between practice environment and turnover intentions (Boudreau and Rheaume, 2024). However, a central contribution of this study is demonstrating that resilience moderated the pathway from practice environment to emotional exhaustion, but did not moderate the pathway from emotional exhaustion to turnover intentions. This asymmetry provides important theoretical insight. Specifically, resilience appears to function primarily as a preventative resource that shapes how environmental demands are internalized, rather than as a corrective mechanism once emotional exhaustion has become established. These findings suggest that resilience operates primarily at the stage of stress response, leading to emotional exhaustion rather than influencing later turnover intentions.

Resilience may therefore buffer nurses from experiencing emotional exhaustion caused by demanding work conditions. However, once emotional exhaustion lasts for an extended period, the influence of resilience on turnover intentions may be less amenable to any interventions aiming at turnover intentions (Rushton et al., 2015). This distinction advances the theoretical understanding of the relationship between resilience, practice environment, emotional exhaustion, and turnover intentions by distinguishing early protective factors from later turnover-related outcomes. Accordingly, resilience-building efforts may be most effective when implemented early, before emotional exhaustion becomes prolonged.

Further examination of the practice environment subcomponents revealed a meaningful pattern; organizational conditions related to staffing and resource support were rated less favorably and demonstrated stronger associations with emotional exhaustion. In contrast, relational aspects of the practice environment, such as nurse–physician relations, were rated more favorably and showed comparatively weaker associations. This pattern suggests that emotional exhaustion among acute care nurses may be more closely linked to organizational conditions such as workload demands and resource support than to professional relational dynamics (Maslach et al., 2018; Rushton et al., 2022). When examined across resilience levels, the association between staffing and resource adequacy and emotional exhaustion was stronger among nurses with lower resilience. Although exploratory, this finding aligns with the moderated mediation results and suggests that resilience may attenuate the psychological impact of demanding practice environment conditions. Nurses with lower levels of resilience may be more susceptible to the effects of staffing constraints and workload demands. These findings underscore the importance of early organizational strategies that reduce exposure to sustained stress while fostering resilience as a protective factor.

In the post-pandemic context, chronic staffing shortages, heightened patient acuity, and moral distress associated with resource limitations all contribute to emotional exhaustion regardless of perceived organizational support (Maslach et al., 2018; Rushton et al., 2022). These broader conditions suggest that improvements in organizational factors alone may not be sufficient to reduce emotional exhaustion without concurrent attention to systemic pressures introduced during and after the pandemic. The moderated mediation framework tested in this study, therefore, provides a conceptual foundation for multi-level strategies aimed at reducing environmental demands, strengthening personal resources, and supporting early identification of emotional exhaustion.

### 4.2 Implications for practice

The findings suggest that nurse retention strategies should be multifaceted and informed by the mechanisms identified in this study. Because resilience buffered only the pathway from practice environment to emotional exhaustion, it may be more effective to implement preventive interventions that reduce exposure to demanding work conditions, rather than those introduced after emotional exhaustion has already developed. Accordingly, hospitals may benefit from adopting coordinated strategies at the organizational, managerial, and individual levels to support retention, rather than relying solely on isolated improvements to the practice environment (ANA, 2025; NASEM, 2024).

At the organizational level, system-wide measures that reduce exposure to sustained stress and protect nurses' well-being are essential ([NASEM], 2024). Strategies such as ensuring adequate staffing, providing coverage for breaks and vacations, and strengthening resource support may help prevent emotional exhaustion (ANA, 2025). Nurse managers are uniquely positioned to identify early signs of emotional exhaustion, foster open communication about mental health, and promote resilience through professional development, structured debriefings, and flexible scheduling (ANA, 2025). These leadership practices not only support resilience but also reinforce a culture of trust and psychological safety (ANA, 2025; [NASEM], 2024).

At the individual level, resilience-building initiatives remain important and may include stress management training, mindfulness programs, peer mentoring, and support for work–life balance (ANA, 2025; Rushton et al., 2021). However, resilience-building efforts should complement organizational improvements rather than substitute for them. Because resilience may buffer stress response but does not fully counteract the effects of prolonged emotional exhaustion, effective retention strategies require concurrent attention to both work conditions and individual support.

### 4.3 Limitations

This study has several limitations. The cross-sectional design restricts the ability to draw causal inference and does not capture changes over time. The use of both random and convenience sampling may limit generalizability of the findings. Additionally, the sample reflected a relatively high proportion of baccalaureate-prepared nurses, which may not represent all acute care nurses in different regions or healthcare systems. Educational preparation may influence factors such as professional autonomy, stress responses, and access to resilience resources.

Self-reported data may introduce recall and social desirability bias, and online participation may lead to selection bias. Although validated instruments were used to assess resilience, practice environment, emotional exhaustion, and turnover intentions, they cannot fully capture the complexity of the concepts. The moderated mediation model may omit confounding variables, and post-pandemic data collection limits broader applicability. While caution is warranted in generalizing the findings, the moderated mediation framework provides a valuable foundation for future longitudinal and mixed-methods studies to build upon these findings and inform targeted interventions.

## 5 Conclusion

This study enhances the understanding of how the practice environment is associated with acute care nurses’ turnover intentions through emotional exhaustion and clarifies the stage-specific role of resilience. Emotional exhaustion emerged as the central mechanism linking work conditions to turnover intentions, while resilience functioned primarily as a preventative moderator influencing stress response rather than subsequent turnover decisions. These findings underscore the importance of early, multilevel intervention strategies that address both work condition factors and personal resilience resources. Hospitals may therefore benefit from coordinated strategies that strengthen the work conditions while also promoting nurses’ resilience and well-being to improve retention.

## Funding

This research did not receive any specific grant from funding agencies in the public, commercial, or not-for-profit sectors.

## CRediT authorship contribution statement

**Elizabeth H. Fraser:** Writing – review & editing, Writing – original draft, Validation, Resources, Project administration, Methodology, Investigation, Formal analysis, Data curation, Conceptualization. **Lingling Zhang:** Writing – review & editing, Methodology, Conceptualization. **Anna E.Schierberl Scherr:** Writing – review & editing, Methodology, Conceptualization. **Sun S. Kim:** Writing – review & editing, Writing – original draft, Validation, Supervision, Methodology, Conceptualization.

## Declaration of competing interest

The authors declare that they have no known competing financial interests or personal relationships that could have appeared to influence the work reported in this paper.
